# Conflicting demands and shifts between policy and intra-scientific orientation during conservation research programmes

**DOI:** 10.1007/s13280-017-0913-y

**Published:** 2017-03-15

**Authors:** Thomas Ranius, Jörgen Rudolphi, Anna Sténs, Erland Mårald

**Affiliations:** 1 Department of Ecology, SLU, Box 7044, 750 07 Uppsala, Sweden; 2 Department of Wildlife, Fish and Environmental Studies, Swedish University of Agricultural Sciences, 901 83 Umeå, Sweden; 3 Department of Historical, Philosophical and Religious Studies, Umeå University, 901 87 Umeå, Sweden

**Keywords:** Conservation biology, Forest biofuels, Policy-science interface, Synthesis, Uncertainties

## Abstract

Conservation scientists must meet the sometimes conflicting demands of policy and science, but not necessarily at the same time. We analysed the policy and intra-scientific orientations of research projects on effects of stump extraction on biodiversity, and found shifts over time associated with these demands. Our results indicate that uncertainties related to both factual issues and human decisions are often ignored in policy-oriented reports and syntheses, which could give misleading indications of the reliability or feasibility of any conclusions. The policy versus intra-scientific orientation of the scientific papers generated from the surveyed projects varied substantially, although we argue that in applied research, societal relevance is generally more important than intra-scientific relevance. To make conservation science more socially relevant, there is a need for giving societal relevance higher priority, paying attention to uncertainties and increasing the awareness of the value of cross-disciplinary research considering human decisions and values.

## Introduction

Research in conservation science has a more complex context than research in basic ecology, since conservation issues are mission driven and often have profound implications for both socio-economic and natural systems (Soulé 1985; Mace 2014). Thus, researchers must work in the interface between policy and science, addressing the sometimes conflicting demands associated with both domains. As scientists, they are trained to value scientific ideals such as objectivity and freedom from political and economic influence in order to maintain scientific integrity (Horton et al. 2015). However, the major objective of conservation science is to provide tools and strategies for preserving biodiversity (Soulé 1985). Thus, many conservation scientists want their research findings to be beneficial in the real world, and the relevant authorities require knowledge that will assist decision-making.

If the aim of the scientific research is mainly to produce solutions for use in policy development it can be described as policy oriented, while if it is aiming for new knowledge, independent of its immediate practical usefulness, it can be described as intra-scientifically oriented (cf. applied and basic science; Heilbron 2003). When scientists are working in the interface between policy and science, different attitudes to these orientations are possible (Pielke 2007), but there is little empirical knowledge about conservation scientists’ behaviour related to these orientations.

Conservation science is often influenced by a sense of urgency and need to act in the face of uncertainty, before all the relevant facts are known (Soulé 1985; Noss 1999). Hence, conservation scientists are often required to evaluate large-scale, long-term consequences of natural processes, human activities and their interactions in highly complex systems, despite the inevitably major uncertainties (Uggla et al. 2016). Even in best case scenarios, likely effects of changes in an ecosystem are only known for small proportions of the taxa that may be affected. Furthermore, different groups of stakeholders often have widely differing goals across the spectrum from pure nature conservation to maximal utilization of natural resources. These conflicts have to be considered by conservation scientists especially if different stakeholders interpret research outcomes from conservation science in conflicting ways, which can sometimes be justified by uncertainties in the available scientific information (Uggla et al. 2016).

Uncertainties arise when making decisions for several reasons. In a conservation context, three major classes of uncertainties have been identified: epistemic (i.e. uncertainties about facts that we could know but do not know), linguistic (i.e. uncertainties about language and meaning of expression), and human decision-related (i.e. uncertainties that arises from subjective human beliefs, values, and judgements) (Kujala et al. 2013). Scientists are mainly trained to address and minimize epistemic uncertainties. However, the other types of uncertainties are also important in communication and decision-making, and thus should be considered by researchers who strive for doing research with a high relevance for society.

A common objective of policy-makers is to set targets and limits for the level of human impact on ecosystems. Such policy decisions typically require guidance from researchers regarding, for instance, sufficient amounts of efforts to meet a certain conservation goal (Wilhere 2008) or the maximum sustainable level of exploitation of a natural resource (Jentoft and Chuenpagdee 2009). To provide such guidance, researchers must understand both the scientific aspects of the problem (involving epistemic uncertainties) and social aspects, including interpretations of “sufficient” and “sustainable” (involving linguistic and human decision uncertainties), and the variations in interpretations of these terms among stakeholders. Thus, this is an example where the two latter types of uncertainties are important and often challenging parts of applied science, which they rarely are in basic science.

There is little knowledge of the behaviour of conservation scientists in research programmes intended to have high societal relevance regarding how they orient their projects towards policy and intra-scientific relevance and how they handle the three sets of uncertainties mentioned above. Therefore, we have addressed these issues by examining two research programmes, spanning eight years in total, funded by the Swedish Energy Agency on effects of harvesting tree stumps (for bioenergy) on biodiversity, chosen partly because the research had a clearly defined remit and time limit. Here we analyse how the policy versus intra-scientific orientation changed during the course of the programmes, and discuss possible adjustments to conservation science practices that could potentially improve the generation of knowledge with high societal relevance and handling of uncertainties.

## Stump harvesting and biodiversity: An issue involving uncertainties

In forested countries, forest biomass could potentially be an important renewable energy source. In Sweden, biomass from forests is already extensively used, but according to governmental goals its use should be further increased (Government Offices of Sweden 2009). The extraction of logging residues (branches, tops, and stumps) after harvesting has been discussed and implemented to varying degrees during at least the last four decades (Edwards and Lacey 2014). In the 1970s, the use of logging residues was expected to mitigate effects of the energy crises, increase employment in rural areas, and improve “hygiene” in forests by reducing amounts of dead wood (e.g. Högström et al. 1978). More recently, it has been advocated to assist efforts to mitigate climate change, but in stark contrast to the 1970s and 1980s there are now major concerns about the low amounts of dead wood in Swedish forests, due to its importance for biodiversity. The Swedish government has formulated a goal to maintain viable populations of all native species, many of which depend on dead wood (de Jong et al. 2012). This means that the most relevant response variable from a conservation perspective is the viability of species at a large spatial scale, but the effect of current management regimes on species viability is expected to be visible only after several decades (Johansson et al. 2016).

To clarify the issues, the Swedish Energy Agency initiated a research programme to assess the sustainability of biofuel utilization in the early 2000s. Initially it mainly focused on slash harvesting, but when several forest companies were ready to start stump extraction as a trial activity the government recognized the need for more knowledge before implementing it at full scale. The programme was intended to increase knowledge of effects of forest biofuel extraction, possible measures to compensate for associated biodiversity losses, and levels of forest biofuel harvest that are acceptable and sustainable (Swedish Energy Agency 2007). Such knowledge is subject to various epistemic, linguistic, and human decision-related uncertainties, which should be considered when attempting to formulate acceptable guidance or feasible strategies.

## Materials and methods

We analysed the policy and intra-scientific orientation in documents from research programmes about consequences of stump extraction on biodiversity. The analysis involved assessing all documents available by 1 April 2016 that were produced in two research programme rounds. Some documents were still not available at that time (including the synthesis for the second round) although the last programme officially ended in 2015. The research programmes (initiated in 2007 and 2011) are the only Swedish programmes including biodiversity-related research on stump harvest. The Swedish Energy Agency and other funding sources have also financed relevant projects outside these programmes, but they were not included in our analysis.

The analysed documents were two calls for proposals, documents concerning 18 specific projects—the applications (one per project), scientific papers generated from the research (15 in total) and reports to the Swedish Energy Agency (one per project)—and one common synthesis report. Only successful applications were included. All analysed projects except one focused on natural science topics. Most scientific publications focused on certain taxa associated with dead wood (fungi: Berglund et al. 2011; beetles: Andersson et al. 2012; Jonsell and Schroeder 2014; Kubart et al. 2016; lichens and/or bryophytes: Caruso and Rudolphi 2009; Caruso et al. 2010, 2011; Hjältén et al. 2010; Rudolphi et al. 2011; Svensson et al. 2013, 2014, 2016a, b) or both cryptogams and beetles (Ranius et al. 2014), but some attention was paid also to soil invertebrates (Taylor and Victorsson 2016). At the end of the scheduled time for each project its leader had to send a report summarizing the outcomes to the Swedish Energy Agency. The synthesis, published and endorsed by the Swedish Energy Agency (de Jong et al. 2012), aimed at summarizing knowledge from the research programme and closely related research, identifying goal conflicts, potential solutions and knowledge gaps.

To analyse the level of policy and intra-scientific orientation of the examined documents, we first developed and discussed possible criteria, second assessed some documents individually, and then compared our assessments and developed the two qualitative, 5-level scales used in the final assessment (Table 1). The intra-scientific orientation scale reflects the level of scientific generality and to what extent the aim of the study was to address intra-scientific questions. The policy orientation scale was based on to what extent the aim of the study was to address policy-relevant questions, especially the more general question about acceptable levels of stump harvesting, considering epistemic, linguistic and human decision-related uncertainties. Most important when assigning scores for the two orientations were the research questions and the conclusions. We analysed the motivations given for why goals or conclusions were relevant. The researchers’ handling of uncertainties in scientific papers, reports and the synthesis was analysed by reading the discussion constituting the background for the conclusions, and in the call and applications by analysing how these were intended to be handled. If motives were clearly related to policy or science, the scores were 3 or higher (Table 1). When the motives were not explicitly stated, it resulted in a scoring of 1 or 2, based on an overall interpretation after reading the whole text. What we described as assisting “policy improvement” typically meant that conclusions were drawn about the consequences of stump extraction on certain aspects of biodiversity, but not necessary that policy issues were explicitly discussed. Sometimes single statements conflicted with the overall impression, and if so they were given minimal weighting. Initially, we assessed each orientation separately, but since we were mainly interested in the relative extents of policy and intra-scientific orientation, we subtracted the policy orientation score from the intra-scientific orientation score to obtain one single policy vs. intra-scientific orientation variable.

**Table 1:** Scales used to score the policy and intra-scientific orientation in examined documents

	Policy orientation	Intra-scientific orientation
5	The main motive was to contribute to policy improvements, addressing uncertainties about acceptable stump extraction levels	The main motive was to contribute to scientific theory
4	The main motive was to contribute to policy improvements, considering acceptable stump extraction levels, but without addressing uncertainties	The main motive was to study a research question associated with a general scientific theory
3	One motive was to assist policy improvement, but not by considering acceptable stump extraction levels	One motive was to describe empirical patterns, and discuss ecological processes influencing them
2	Assisting policy improvement not explicitly described as a motive, but the generated knowledge may still be useful for this	The project increased knowledge about the biology (like habitat associations) of organisms, but not about processes influencing observed patterns
1	No relation to policy improvement	The project only addressed system-specific questions, ignoring research questions beyond the conditions in a specific situation or study area

## Results

**Fig. 1:**
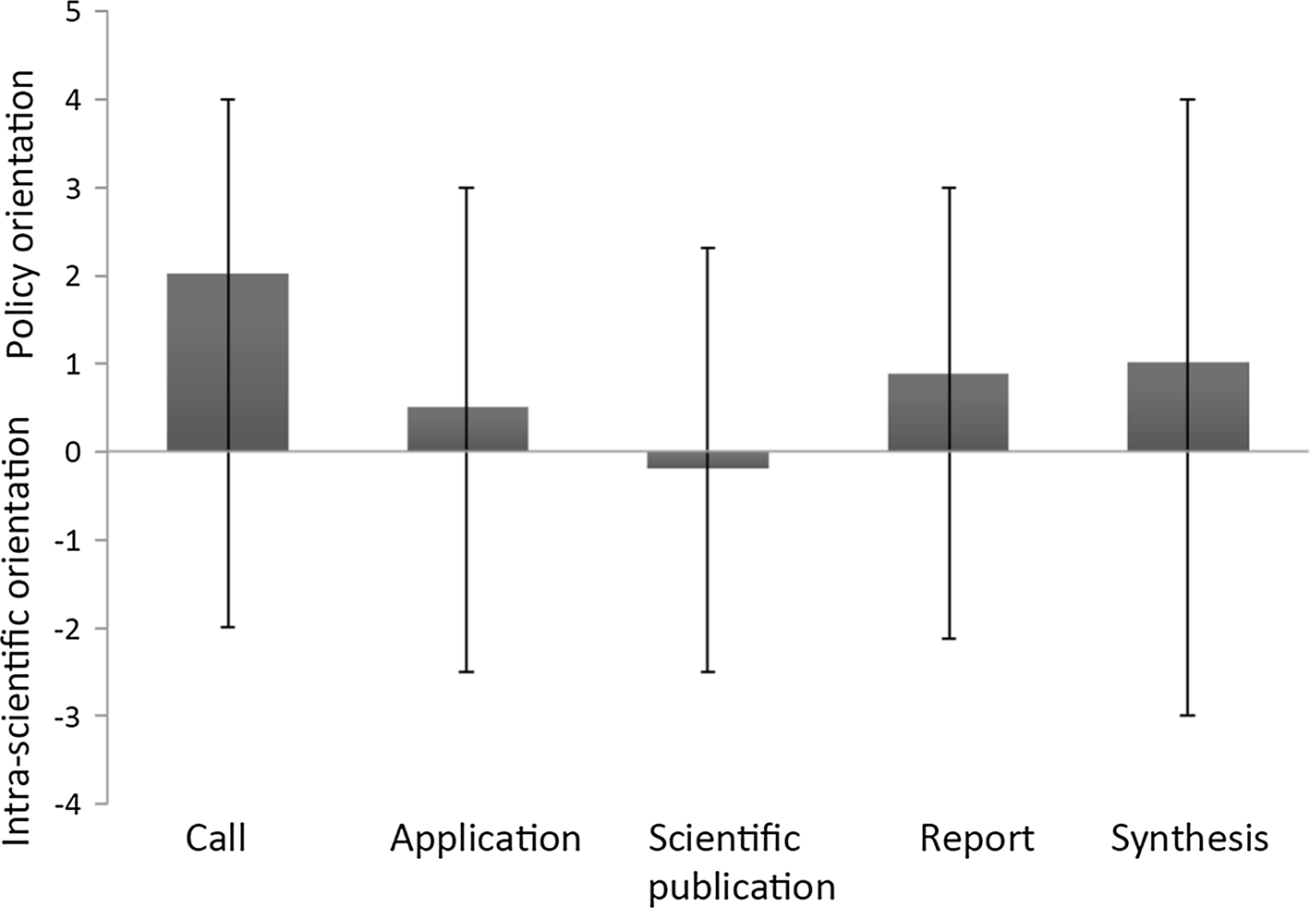
The level of policy and intra-scientific orientation in the examined documents (see text for details) from two research programmes on effects of harvesting tree stumps on biodiversity. The bars show the policy versus intra-scientific orientation (i.e. policy orientation minus intra-scientific orientation), the upper whisker the policy orientation and the lower whisker the intra-scientific orientation (multiplied with −1). All are mean values for categories of documents as measured by the scale in Table 1

The analyses showed that there was a shift between policy and intra-science orientation over time during the research programmes (Fig. 1). In the first call (in which more aspects than only biodiversity were considered), three goals were formulated: (i) “Methods for efficient and sustainable forest management for increased production of forest bioenergy should be developed”, (ii) “Strategies and methods for energy production from intensively managed forest should be developed”, and (iii) “The level for acceptable, sustainable outtake of forest biofuels should be clarified” [translated from Swedish] (Swedish Energy Agency 2007, p. 3). Thus, all goals clearly stated a demand for policy relevant research. The third goal implied a need for quantitative data on critical thresholds that could be used by policymakers. However, there were clearly shifts towards intra-scientific orientation in the accepted applications and the following scientific publications. In all except one application and one report, the researchers avoided attempts to quantify acceptable levels of biofuel harvesting, which was a key policy issue. According to this application, data would be analysed to provide “thresholds for how many stumps that should be left after stump harvest to avoid negative impacts on wood living organisms” (Swedish Energy Agency, Dnr 2012-002817), while in the report (which was for a different project), it was stated that “an outtake of 50% of the available bioenergy wood should be completely acceptable from a biodiversity point of view since it gives small negative effects on wood-living flora and fauna” [translated from Swedish] (final report to the Swedish Energy Agency, project 35217-1).

**Fig. 2:**
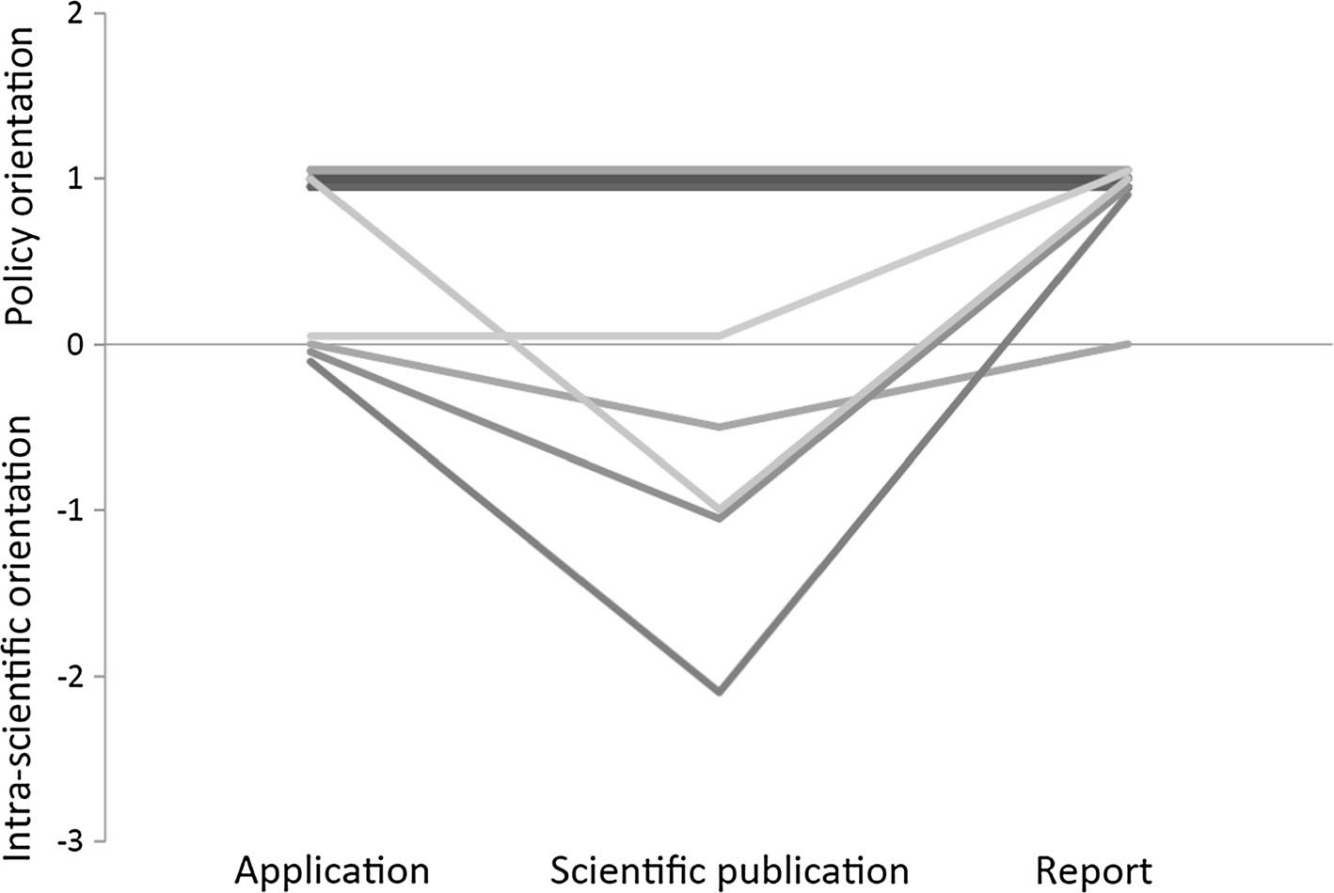
Changes in the policy versus intra-scientific orientation (i.e. policy orientation minus intra-scientific orientation as measured by the scale in Table 1) during the courses of the eight research projects for which data for all three stages were available. Data from two research programmes on effects of harvesting tree stumps on biodiversity

The scientists’ reports for the Swedish Energy Agency were, on average, more policy-oriented than the scientific papers. However, there was large variation among the projects; a constant level of policy versus intra-scientific orientation was maintained in some of them throughout the application, scientific publication and report sequence, while in others the scientific papers produced were much more intra-scientifically oriented than the applications and reports (Fig. 2). An example of the latter was a project which according to the application aimed at generating “knowledge that can serve as a basis for recommendations about which regions where stump harvesting may be conducted considering species diversity of mosses, lichens, fungi and insects” (Swedish Energy Agency, project 2007-02686), while the aim of one of the resulting scientific papers was to “increase the understanding of metapopulation dynamics of species that occupy patches that appear, change over time, and finally deterministically disappear” (Caruso et al. 2010). The synthesis included an evaluation of the sustainable level of biofuel outtake (in a chapter entitled “Synthesis—can we increase the outtake of forest bioenergy without negative consequences for the environment?” [Translated from Swedish]) (de Jong et al. 2012, pp. 157–183), and hence had a high level of policy orientation (Fig. 1). It suggested that stump harvesting would substantially compromise biodiversity goals if practised at ≥20% (but not ≤10%) of all clear cuts. The results were related to current Swedish environmental policy, but not to potential conflicts due to diverging values among key stakeholders.

The research programme studied here could be described as policy-science cycles divided into two phases, in which the scientists work in different ways. First, there was a scientific research phase (spanning the time between the application and submission of scientific papers), in which the projects generally became more intra-scientifically oriented. Second, there was a synthesis phase in which the reports and synthesis were prepared and the research returned to a higher level of policy orientation (Fig. 1).

In the scientific research phase, all presentations of results followed a similar format, probably because all the researchers engaged in the projects shared a goal to publish original research papers in peer-reviewed natural science journals. In the synthesis phase, the presentations were more variable. For instance, about half of the reports were divided into *Results*, *Discussion* and *Conclusion* sections, while the others had no *Discussion* section, which hindered comprehension of connections between the results and conclusions or of any uncertainties in the interpretations. Potential epistemic uncertainties were due to the fact that the studies often represented snapshots from certain habitats or study sites, while more general interpretations were drawn. For instance, a conclusion in a report was that “stump harvesting is not expected to significantly affect the status of fungi of conservation interest”, based on a survey of fungi in stumps and logs (Swedish Energy Agency, project 35208-1). Also linguistic uncertainties may occur due to that expressions such as “species of conservation interest” can be interpreted in different ways. Some of the reports gave an impression of preliminary drafts, possibly because most had been submitted before associated scientific papers.

The synthesis had a chapter about the consequences of biofuel harvests on biodiversity, which carefully cited the sources (de Jong et al. 2012, pp. 113–156), including not only publications, but also reports and oral communications from project leaders. It also included a chapter presenting assessments of the consequences of different levels of biofuel harvesting, but it was not reported how these assessments were related to theoretical or empirical research, and consequently epistemic uncertainties were not discussed (de Jong et al. 2012, pp. 157–183). The chapter about biodiversity was written by one single author who also was leader for one of the research projects, while the chapter with assessments was written by seven authors with a more varying background regarding scientific work and conservation biology.

## Discussion

### The policy-science cycle

In the scientific research phase of the research programmes, many scientists seemed to strive for a more science-oriented direction, different from the policy-orientation of the calls (Fig. 1). This is consistent with a previously reported process called ‘academic drift’, whereby knowledge intended to be useful gradually loses close ties to practice and becomes more closely integrated with scientific knowledge (Harwood 2010). In the synthesis phase, there was a shift in the opposite direction, back towards a policy orientation (Fig. 1), because the Swedish Energy Agency demanded reports and a synthesis. Such a process has been referred to as ‘epistemic drift’, whereby researchers embrace values from ideological systems external to science, for example industry or politics, and pay greater attention to the potential uses of their activities (Elzinga 1985; Kaiserfeld 2013). In these research programmes, it was obvious which products of the projects were generated in different parts of the policy-science cycle, but in many other cases it is difficult to define cycles because several cycles may be interacting (cf. Lidskog 2014), or scientific and policy issues may interact more continuously over time (Prescott and Weese 2014). Nevertheless, we believe that even in such cases the phases and drifts we have described and discuss below still occur.

Organizing research programmes according to a policy-science cycle, as described here, allows scientists to address research questions in scientific papers relatively independently and still directs the projects towards issues of interest for the commissioning authority in the synthesis. However, according to our experience there is no synthesis phase in many research programmes, or it is weak. Our results indicate that in the focal programmes this would have led to the end products being mainly scientific papers addressing issues that in many cases considerably differed from the questions posed by the commissioning authority (Figs. 1, 2).

One reason for the lack of syntheses is that they are typically supposed to be written as the last activity in research projects, and if funding bodies do not strongly encourage such activities there is a risk that too little time and resources are left for this activity. There were a few examples of that also in the research projects we analysed; there was one project that according to the application should organize a workshop for scientists and stakeholders where common conclusions and recommendations were formulated, but this never took place (Swedish Energy Agency, project 36135-1). Furthermore, two projects out of three intending to put their biological studies in a larger perspective including cost efficiency never included any economic aspects in their projects (Swedish Energy Agency, project 36135-1 and 35217-1). Thus, it is not enough only to state the applied aspects in the calls, but policy-related outcomes and activities that really take place should be encouraged.

We found wide variation in the orientation of the scientific papers and both the formats and contents of the reports and synthesis. We believe that the societal relevance of conservation science can be enhanced by increasing awareness and training among both scientists and research funders of the importance of maintaining focus on societal dimensions of the focal issues during the scientific research phase and communicating and integrating uncertainties in the synthesis phase. We discuss these two aspects below.

### Scientific and societal relevance of research in conservation science

We found that the scientific publications generated from the examined projects spanned a broad range of positions along the policy versus intra-scientific scale (Fig. 2). Those that were most intra-scientifically oriented had a substantially different position from the corresponding applications and reports, while more policy-oriented papers had similar positions to the corresponding applications and reports. This difference among the papers probably reflect different attitudes among authors; our impression is that some ecologists are mainly striving for societal relevance and other for intra-scientific relevance. Intra-scientific relevance is usually achieved by developing and testing general ecological theory, while societal relevance is achieved by developing or testing management and policy options in terms of their broad effects on biodiversity conservation and potentially conflicting (e.g. economic) goals.

In projects related to stump extraction, the societal relevance was increased by considering several groups of organisms in the same study (since persistence of overall biodiversity is a main goal), by performing studies in various regions, by cross-disciplinary approaches, or by assessing effects at a wide range of spatial and temporal scales. These approaches cover a larger proportion of aspects that may influence decisions than narrower approaches. To obtain a high overall societal relevance of a research programme requires large components that are broad and policy-oriented, but more narrow projects of high scientific relevance may be important as complements. We found some examples of the latter type of projects in our analysis, in which ecological theory was utilized to formulate general questions about, for instance, facilitation of lichen colonization and small-scale colonization-extinction patterns. However, since no applications were clearly intra-scientifically oriented (Fig. 2), this was probably not resulting from a strategy at a research programme level.

In basic ecological research, it is sufficient to strive solely for a high level of intra-scientific relevance, but it seems that this is also done by many researchers in applied ecology, including conservation science. We argue that in applied research it is, by definition, more appropriate to strive for societal relevance. However, we still believe that applied research usually do better when closely coupled to ecological theory, since it improves predictions, framing and planning of research, and communication with other scientists (Driscoll and Lindenmayer 2012). Hence, ecological theory (e.g. island biogeography, metapopulation and demographic theory) played a central role in the early history of conservation biology (Kendall 2015), but its input has been less apparent in more recent papers in conservation journals (Fazey et al. 2005). One reason for the weak interest in ecological theory among some conservation scientists may be that predictions rooted in theory are often found less useful because of their deficiencies in the spatial, temporal or taxonomic contexts addressed (Driscoll and Lindenmayer 2012). However, we argue that this provides a strong reason for maintaining the focus on ecological theories in conservation science, in order to test the theory in contexts of high societal relevance, i.e. considering response variables and spatial scales relevant according to policy, for instance long-term biodiversity conservation at larger spatial scales or maintenance of important ecosystem services.

### Integrating and communicating uncertainties

Epistemic uncertainties occur in both the scientific research and synthesis phases, but pose bigger challenges in the synthesis phase. This is because scientists are free to formulate research questions as they see fit during the research phase, and hence they can avoid issues associated with large uncertainties. For instance, scientific publications may consider only relatively small spatial and temporal scales, avoiding discussions about larger scales. However, this is not an option in the synthesis phase, at least if it would mean neglecting the issues of highest policy-relevance, which often include large spatial scales (cf. Stevens et al. 2007). If important aspects have not been considered in scientific studies, the synthesis may conclude that there is a knowledge gap. Another option may be to use expert knowledge in a systematic manner, e.g. by the Delphi technique (Mukherjee et al. 2015).

If the intention is to develop and evaluate policy alternatives, human decision-related and linguistic uncertainties should also be considered, at least in some phase of a research programme. This is because scientific knowledge is only one of several factors influencing policy-making (Rose 2015). Furthermore, the terminology is only to some extent the same among scientists and stakeholders, and some terms have a different meaning in different contexts (cf. Star and Griesemer 1989). With a better understanding of human decision-making processes and the variation in human values, more relevant scientific knowledge can be developed. One possible approach to obtain such an understanding are collaborative processes, in which scientists and various stakeholders together identify and contextualize uncertainties, conflicting goals, and divergent values (Norton 2005; Balint et al. 2011). In this way, the variety in values among stakeholders is revealed, which facilitates the development of corresponding policy options. This makes it easier to follow Pielke’s (2007) recommendation that scientists should present, or ‘broke’, several alternative options for policy-makers. Thus, the scientific knowledge can be presented in a more useful way.

## Conclusions: From conservation biology to conservation science

The present behaviour of conservation scientists is a result of culture, and especially universities, funding sources, and leading scientists are able to modify and in the long term change this culture. Modern conservation biology took important developmental steps about 40 years ago (Noss 1999). Many conservation scientists were initially trained in pure ecology, zoology, or botany (Noss 1999) and their scientific ideals are currently rooted in these disciplines, even though their personal motivation may be to contribute to biodiversity conservation. However, conservation problems have wider societal contexts than purely biological or ecological problems, and their solution requires broader competence, including understanding of relevant aspects of political sciences, economics, and humanities (Jacobson and McDuff 1998). This has become pronounced to an increasing extent over time, since conservation science has changed from focusing on the protection of intact natural habitats to including a wider variety of attitudes to nature, also recognizing the dynamic relationship between people and nature (Mace 2014). As more scientists receive a broader education in conservation science, the conservation science community will become more self-confident to develop its own research ideals. To justify the existence of conservation science as a distinct discipline, it has to be relevant for society. Therefore, societal relevance should become a more important criterion than intra-scientific relevance when evaluating conservation science research programmes. It is also important for conservation scientists to be good at understanding, integrating and communicating different types of uncertainties. In addition, more attention should be paid to cross-disciplinary research and synthesis efforts.

To date, conservation scientists have often avoided complex issues involving large uncertainties, and focused instead on more tightly delimited problems. However, publicly responsible conservation scientists sometimes handle complex problems that are known to involve large uncertainties, even the so-called ‘wicked’ problems (Balint et al. 2011; Game et al. 2013). Some stakeholders may have unrealistic expectations that scientists should be able to provide simple answers to complex problems. In such situations, it is important for scientists not to deliver what the stakeholders want but what they need, by clearly expressing the complexities and uncertainties involved, and avoiding temptations to promise too much.
